# Hybrid EEG Feature Learning Method for Cross-Session Human Mental Attention State Classification

**DOI:** 10.3390/brainsci15080805

**Published:** 2025-07-28

**Authors:** Xu Chen, Xingtong Bao, Kailun Jitian, Ruihan Li, Li Zhu, Wanzeng Kong

**Affiliations:** School of Computer Science and Technology, Hangzhou Dianzi University, Hangzhou 310018, China; 22051040@hdu.edu.cn (X.C.); 21052001@hdu.edu.cn (X.B.); 22050228@hdu.edu.cn (K.J.); 22050229@hdu.edu.cn (R.L.); kongwanzeng@hdu.edu.cn (W.K.)

**Keywords:** EEG-based mental attention states decoding, cross-session classification, hybrid feature learning, feature selection, brain computer interface

## Abstract

Background: Decoding mental attention states from electroencephalogram (EEG) signals is crucial for numerous applications such as cognitive monitoring, adaptive human–computer interaction, and brain–computer interfaces (BCIs). However, conventional EEG-based approaches often focus on channel-wise processing and are limited to intra-session or subject-specific scenarios, lacking robustness in cross-session or inter-subject conditions. Methods: In this study, we propose a hybrid feature learning framework for robust classification of mental attention states, including focused, unfocused, and drowsy conditions, across both sessions and individuals. Our method integrates preprocessing, feature extraction, feature selection, and classification in a unified pipeline. We extract channel-wise spectral features using short-time Fourier transform (STFT) and further incorporate both functional and structural connectivity features to capture inter-regional interactions in the brain. A two-stage feature selection strategy, combining correlation-based filtering and random forest ranking, is adopted to enhance feature relevance and reduce dimensionality. Support vector machine (SVM) is employed for final classification due to its efficiency and generalization capability. Results: Experimental results on two cross-session and inter-subject EEG datasets demonstrate that our approach achieves classification accuracy of 86.27% and 94.01%, respectively, significantly outperforming traditional methods. Conclusions: These findings suggest that integrating connectivity-aware features with spectral analysis can enhance the generalizability of attention decoding models. The proposed framework provides a promising foundation for the development of practical EEG-based systems for continuous mental state monitoring and adaptive BCIs in real-world environments.

## 1. Introduction

William James said that attention is the acquisition by the mind, in a clear and vivid form, of one out of what seems to be several simultaneously possible objects or trains of thought. Focalization, concentration, and consciousness are of its essence [1]. Research in the educational field indicates that students with greater concentration in the classroom have an enhanced ability to comprehend the taught material. Elevated attention is crucial for maintaining the quality of teaching [2,3]. Research within the transportation sector reveal a strong correlation between driver distraction and the incidence of close-range accidents. In the realms of education quality and traffic safety, accurately identifying attention is essential [4].

Accurately classifying human mental attention states has long been a topic of interest in cognitive neuroscience and brain–computer interface (BCI) research. In early studies, attention detection was often coupled with computer vision technologies, such as object recognition and gaze tracking. For example, Cheng et al. detected attention states by analyzing the position and rotation angle of the nostrils, achieving 85.8% accuracy using support vector machines (SVMs) [5]. However, these behavior-based approaches are highly susceptible to individual habits and external conditions, limiting their generalizability and robustness across time and subjects.

Given the limitations of behavioral approaches, researchers have increasingly turned to neurophysiological methods to assess attention, each offering distinct advantages and limitations. Functional magnetic resonance imaging (fMRI) provides superior spatial resolution and anatomical specificity for localizing attention-related brain networks [6], but its limited temporal resolution and requirement for controlled environments restrict its applicability for real-time attention monitoring. Functional near-infrared spectroscopy (fNIRS) offers a compromise between spatial resolution and portability [7], though its penetration depth is limited to cortical layers. Positron emission tomography (PET) provides excellent spatial resolution [8] but involves radiation exposure and is unsuitable for repeated measurements. In contrast, electroencephalography (EEG) offers millisecond temporal resolution that is optimal for capturing the dynamic neural oscillations underlying attentional processes [9]. Despite its limited spatial resolution and primarily scalp-level signal capture, EEG’s non-invasive nature, cost-effectiveness, and portability make it uniquely suited for developing practical attention monitoring systems that can be deployed in naturalistic environments. Consequently, many researchers have adopted EEG-based approaches for attention state classification, leveraging its temporal advantages to capture attention-related neural dynamics. Early EEG studies demonstrated promising results. For instance, Liu et al. used EEG power spectral features to classify two mental states and achieved 76.82% accuracy in an inter-subject setting using SVM [10]. Niu et al. explored multiple feature extraction methods—wavelet transform (WT), empirical mode decomposition (EMD), and local mean decomposition (LMD)—and reported accuracies above 89% for binary attention classification using SVM [11]. Mohamed et al. extracted time- and frequency-domain features from 86 participants and achieved 84% accuracy in three-class attention classification within a session [12]. Similarly, Chen et al. integrated time, frequency, and nonlinear dynamic features, achieving 85.05% ± 6.87% accuracy [13], while Suhail et al. fused wavelet, entropy, coherence, and phase-locking value (PLV) features, reaching an average accuracy of 88.97% across three attention-related tasks [14]. Despite promising results, most existing studies are limited in several key aspects. First, classification under inter-subject and cross-session conditions remains challenging due to significant inter-individual variability and session-related fluctuations. For example, Hu et al. integrated correlation-based feature selection with K-nearest neighbors (KNN) and achieved 80.84% accuracy for three-class attention recognition in a cross-session intra-subject setting [15], but performance degradation under such conditions remains a major obstacle for real-world applications.

Although deep learning methods such as convolutional neural networks (CNNs), deep belief networks (DBNs), and long short-term memory (LSTM) networks have shown great potential in learning hierarchical EEG features [16], they are often limited by high data requirements, poor generalizability in cross-subject/cross-session scenarios, and lack of interpretability. For instance, Wang et al. employed CNNs on STFT-based EEG features and achieved over 94% accuracy in personalized attention detection [17], while Hassan et al. and Toa et al. employed CNN-LSTM and convolutional attention memory neural networks (CAMNN), reaching accuracies of 89% and 92%, respectively [18,19]. Yet, these models are typically optimized for individual-specific data and may not scale well across populations or recording sessions.

In summary, EEG-based attention decoding still faces several critical challenges that limit its practical applicability. Existing models often lack robustness in inter-subject and cross-session scenarios, making it difficult to generalize across individuals and recording conditions. While previous studies have explored multi-domain features—including time, frequency, and nonlinear metrics—very few have incorporated diverse brain connectivity measures into the feature space [10,12,13,14]. Furthermore, many reported that high accuracies are achieved under tightly controlled within-session or subject-specific settings, which undermines their external validity. Although deep learning approaches show considerable potential, they are often constrained by limited interpretability, insufficient performance in low-resource or few-shot conditions, and instability across diverse experimental contexts.

The main contributions of this work are summarized as follows:

- We propose a hybrid EEG feature learning framework for cross-session mental attention state classification, which integrates short-time Fourier transform (STFT) features with multiple brain connectivity representations, including functional and effective connectivity, enhanced through a two-stage feature selection strategy.
- We systematically investigate the influence of key signal segmentation parameters—such as window length and overlap ratio on classification performance—providing practical insights for EEG-based system design.
- Extensive experiments on two benchmark EEG datasets demonstrate the effectiveness of our method, achieving average accuracies of 84.3% and 96.61% for intra-subject cross-session classification, and 86.27% and 94.01% for inter-subject classification on Dataset 1 and Dataset 2, respectively, validating its generalizability under realistic conditions.

We structure the remainder of this paper as follows. Section 2 presents the description of our dataset and indicates the implementation of our proposed method. The experimental results are revealed in Section 3. Section 4 presents high distinctive brain connectivity feature and other key features, along with an explanation of the inflated result obtained by Çiğdem İnan Acı et al. [20].

## 2. Related Works

### 2.1. EEG-Based Mental Attention State Experiments

Electroencephalography (EEG) has been widely adopted for investigating human mental attention states due to its high temporal resolution and non-invasive nature. Over the past decade, numerous experimental paradigms have been developed to elicit and classify various attention states, ranging from sustained attention and vigilance to mind-wandering and drowsiness. These paradigms typically fall into three categories: event-related tasks, continuous cognitive engagement, and naturalistic stimuli-based protocols.

In event-related designs, participants are often exposed to time-locked stimuli, such as the oddball paradigm or Go/No-Go tasks, to provoke attentional shifts and assess selective attention via event-related potentials, particularly the P300 and N200 components [21]. While effective, such paradigms may not reflect real-world cognitive fluctuations. To overcome this limitation, continuous task-based settings have been proposed, including sustained attention to response tasks and simulated driving or monitoring tasks, where fluctuating attention levels are annotated based on performance metrics and physiological features [20,22].

More recently, naturalistic and ecologically valid paradigms have gained attention, especially in educational and workplace settings. These include watching online courses, attending lectures, or interacting with digital content, with EEG signals labeled using a combination of task type, self-reports, and behavioral validations. Such studies often incorporate sliding window segmentation, multimodal recordings, and personality trait profiling to enrich the contextual understanding of attention. Publicly available datasets, such as MEMA [23], have further accelerated benchmark development for attention state classification under varying levels of cognitive demand.

### 2.2. Feature Engineering-Based Mental Attention State Classification

Feature engineering remains a fundamental approach in EEG-based mental attention classification, particularly in traditional machine learning pipelines where interpretability and computational efficiency are critical. This approach involves extracting meaningful and discriminative features from raw EEG signals to characterize distinct attention states such as focused, unfocused, or drowsy. These features are then fed into classifiers such as SVM or KNN to perform state recognition.

Commonly used features fall into three main categories: time-domain, frequency-domain, and nonlinear dynamic features. Time-domain features include statistical descriptors (e.g., mean, variance, kurtosis), as well as signal mobility and complexity indicators such as Hjorth parameters [24]. Frequency-domain features are derived via power spectral density (PSD) or band-specific energy which have shown strong correlation with attentional engagement [25]. Nonlinear dynamic features, such as sample entropy, fractal dimension, and Lyapunov exponents, capture the intrinsic complexity of EEG dynamics during varying cognitive loads [26]. Additionally, spatial–spectral features such as differential entropy and common spatial patterns have been extensively employed in multi-class attention classification tasks [27].

More recently, brain network features have attracted growing attention, where EEG signals are represented as functional connectivity networks or topological graphs. Metrics such as PLV, coherence, and graph-theoretical measures have been leveraged to capture inter-regional interactions relevant to sustained attention and mental fatigue [28]. These network-level features provide a complementary perspective to traditional node-based descriptors, enhancing robustness against local noise or variability.

In many studies, features from different domains are fused into hybrid feature sets to improve classification performance. However, this often leads to high-dimensional feature spaces, which may introduce redundancy and impair generalization, especially with limited training data. Consequently, feature selection techniques have become essential to retain informative features and mitigate overfitting. Despite these advances, challenges remain in balancing feature diversity with dimensional efficiency, as well as in establishing generalized feature representations across subjects and sessions.

### 2.3. Deep Learning-Based Mental Attention States Classification

Mental attention state classification using deep learning techniques, particularly through EEG signals, has gained considerable attention due to its ability to decode cognitive states with high accuracy. Early work in this area focused on single-session classification tasks, where deep learning models like CNNs were applied to classify mental attention states based on EEG data from a single session. These models excelled in learning the spatial features of EEG signals, achieving classification accuracy between 84% to 92% for recognizing different mental states [19,29]. However, the generalization of these models was limited when applied to new subjects or sessions. This limitation arises from the variability in EEG signals across individuals and sessions, which often leads to overfitting on specific subject data, reducing the model’s robustness.

To overcome the limitations of single-session classification, more recent approaches have shifted towards cross-subject classification. In this approach, deep learning models are trained on data from multiple subjects, aiming to learn features that generalize across individuals. Hybrid architectures, such as CNN-LSTM models, have been explored to capture both spatial and temporal features of EEG signals. These models have shown promising results in classifying mental attention states across subjects, achieving classification accuracy ranging from 75% to 91.45% in cross-subject two-class validation tasks [30,31]. However, the challenge of variability in EEG signals across different individuals still persists. Large and diverse datasets are required to train such models effectively, and the computational demands increase significantly as model complexity grows.

The final progression in this research area is the move towards cross-session classification. In this context, the temporal dynamics of attention states are taken into account, where attention can fluctuate across different time periods due to factors like fatigue or cognitive load. To address this, models based on recurrent networks, such as LSTM and Transformer-based models, have been introduced. These models capture long-term dependencies in EEG signals and have shown to significantly improve the robustness of mental attention states classification across sessions, achieving 7-fold balanced accuracy of up to 64% [32]. Despite these advancements, the major challenges of requiring large amounts of labeled training data, high computational costs due to millions of model parameters, and the lack of interpretability remain. As deep learning models become more complex, the need for transparency in understanding the decision-making process becomes critical, particularly in clinical and cognitive state monitoring applications.

## 3. Materials and Methods

**Figure 1:**
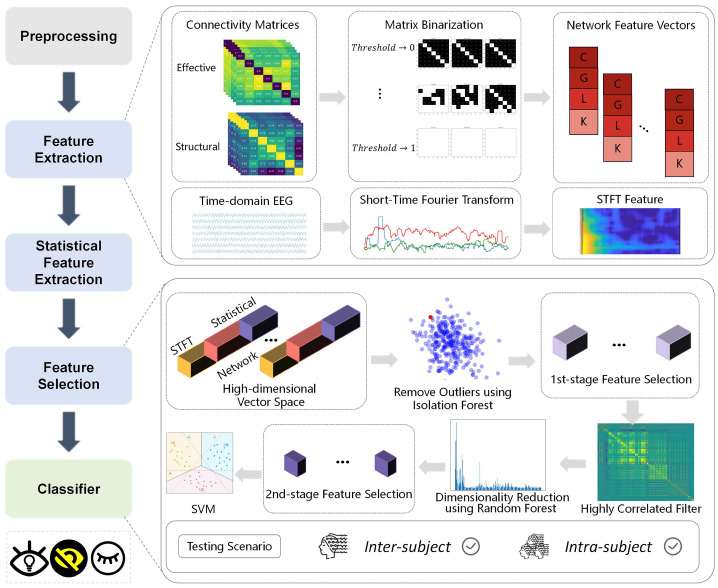
The overall framework of our proposed method applied to mental attention state three-class classification task. The symbols used in the Network Feature Vectors represent the following metrics: C-clustering coefficient, G-global efficiency, L- local efficiency, and K-node degree.

This section describes the dataset, preprocessing pipeline, feature extraction and selection procedures, and the classification framework used in our study. An overview of the proposed method is illustrated in Figure 1. The workflow consists of five main stages: preprocessing of raw EEG signals; feature extraction including connectivity matrices (effective and structural), matrix binarization, network feature vectors, time-domain EEG analysis, and STFT feature extraction; statistical feature extraction combining STFT and network features into a high-dimensional vector space; outlier removal using isolation forest; and feature selection employing a two-stage approach with dimensionality reduction using LDA and random forest, culminating in SVM classification. The framework supports both inter-subject and intra-subject testing scenarios, demonstrating the robustness and generalization of our method across different validation conditions.

### 3.1. Dataset Description

Dataset 1: The publicly available EEG dataset is released by Çiğdem İnan Acı et al. [20]. The recordings were collected from five participants performing a low-intensity, computer-simulated train operation task designed to induce varying levels of mental attention state. Three mental states were labeled and analyzed: focused attention, unfocused but awake, and drowsiness.

Each experiment consisted of a continuous 30 min EEG recording, segmented into three consecutive 10 min blocks corresponding to the three attention states. A total of 34 experimental sessions were conducted, with the first four participants completing 7 sessions each and the fifth participant completing 6. To eliminate adaptation bias, the initial two sessions from each participant were designated as familiarization trials and were excluded from subsequent analyses. Additionally, one session from the fourth participant was discarded due to incomplete data (<30 min). Ultimately, our analysis incorporated five valid sessions each from the first three participants and four valid sessions each from the remaining two participants.

**Figure 2:**
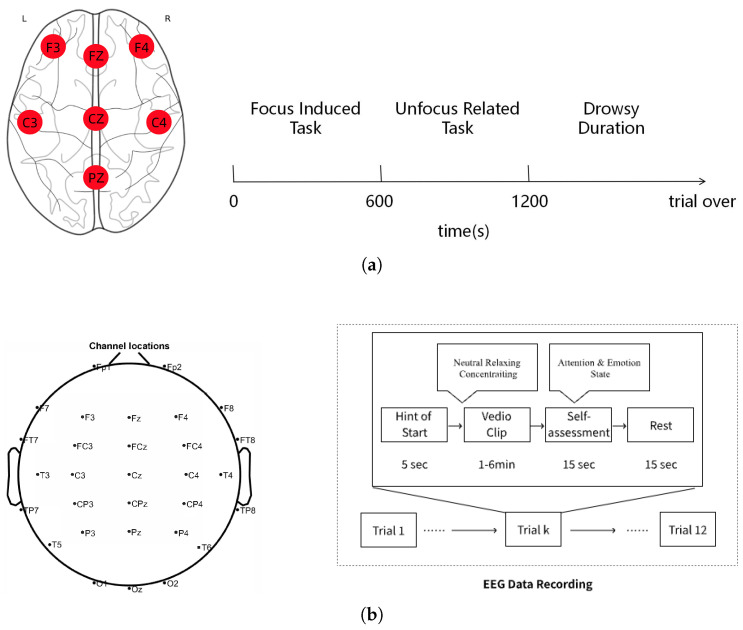
Overview of experimental protocol used for EEG data collection: (**a**) Dataset 1; (**b**) Dataset 2.

EEG data were recorded using a modified Epoc EEG headset with electrodes positioned according to the international 10–20 system at eleven sites: F3, Fz, F4, C3, Cz, C4, T3, T4, T5, T6, and Pz (shown in Figure 2a). As four electrodes (T3, T4, T5, and T6) were dedicated to current injection and reference signal acquisition and thus not used for recording neural activity, only seven electrodes (F3, Fz, F4, C3, Cz, C4, and Pz) were retained for subsequent analysis.

Dataset 2: The publicly available MEMA dataset was released by Liu et al. [23]. EEG signals were collected from 20 participants who completed a standardized cognitive task battery designed to elicit three distinct attention states: neutral, relaxing, and concentrating. Each participant completed 12 trials, each containing three tasks in fixed order across four blocks. To validate subjective attention state labeling, self-reported mental and emotional status was recorded after each task segment. All trials followed a consistent structure, ensuring temporal alignment and repeatability for machine learning research on attention classification.

Each experimental trial consisted of a 5 s instruction cue, followed by a cognitive video task, a 15 s self-assessment, and a 15 s resting interval (shown in Figure 2b). The three task types were as follows: neutral—passive viewing of a 1 min black screen; relaxing—a 5 min nature scene with background music to evoke a relaxed state; and concentrating—a 5 min online machine learning course video, followed by comprehension questions to enforce cognitive engagement. Tasks were always presented in the same order within each trial to minimize intra-session variability. Participants were allowed to extend their resting period voluntarily to reduce fatigue accumulation. In total, over 14 h of multichannel EEG data were collected across all participants.

EEG signals were recorded using the ZhenTec-NT1-32 wireless EEG system with 32 active channels placed according to the international 10–10 electrode layout. Recordings used CPz as reference and FPz as ground, with a sampling rate of 500 Hz and electrode impedance maintained below 20 kΩ.

### 3.2. Data Preprocessing

Due to the high susceptibility of EEG signals to external environmental noise and artifacts, preprocessing is essential to enhance the signal-to-noise ratio prior to feature extraction. The data preprocessing pipeline consisted of band-pass filtering followed by Independent Component Analysis (ICA), applied consistently to both datasets. Previous studies have identified that, during wakefulness, brain-generated EEG signals predominantly occupy the theta (4–8 Hz), alpha (8–12 Hz), and beta (12–30 Hz) frequency bands. Since the optimal frequency band for classification was initially undetermined, the raw signals were filtered into these three conventional bands separately. Delta waves (below 4 Hz), which are typically dominant during sleep stages, were excluded from analysis due to their limited relevance to the current wakefulness-focused study [33].

ICA aims to recover statistically independent source signals from observed sensor data, which are assumed to be linear mixtures of these sources [34]. By establishing a new linear coordinate system, ICA maximizes statistical independence among extracted components, effectively isolating artifacts such as eye blinks and muscle activity. To further suppress noise and improve signal fidelity, the decomposition of ICA was performed using the EEGLAB toolbox (version 2023.1, https://sccn.ucsd.edu/eeglab/) in MATLAB (R2022b; MathWorks, Natick, MA, USA), employing the default algorithm ‘runica’ [35].

For both datasets, the preprocessed EEG signals were segmented using a sliding window approach with a window length of 5 s and a step size of 6 s. This resulted in overlapping samples that capture dynamic temporal features while maintaining computational efficiency. Given the sampling rate of 128 Hz in Dataset 1 and 500 Hz in Dataset 2, each resulting segment contained 640 and 2500 data points, respectively. In Dataset 1, each mental state (focused attention, unfocused but awake, and drowsiness) lasted 10 min per session, whereas in Dataset 2, each trial consisted of three tasks corresponding to neutral (1 min), relaxing (∼5 min), and concentrating (∼5 min) conditions, repeated over 12 trials per participant (four per condition). To reduce the influence of transient effects typically observed at the onset of cognitive states, the first 4 s of each state were excluded prior to segmentation in both datasets.

### 3.3. Feature Extraction

#### 3.3.1. Brain Connectivity

All brain connectivity analyses were conducted using the HERMES toolbox [36], with default parameter settings, unless otherwise specified. Connectivity metrics were computed within the theta (4–8 Hz), alpha (8–12 Hz), and beta (12–30 Hz) frequency bands.

- Structural Brain Connectivity Phase Locking Value (PLV) and Phase Lag Index (PLI) are widely employed metrics for quantifying phase synchronization between neural signals. PLV measures the consistency of phase locking between two time series within a given frequency band and temporal window [37], while PLI quantifies the asymmetry of phase differences, reflecting the directional consistency of phase lead or lag between signals [38].
  Let $\Delta \phi_{rel}\left(t\right)$ denote phase differences between two time series, and the PLV is defined as follows:
  (1) $$PLV=\left|\langle e^{i\Delta \phi_{rel}\left(t\right)}\rangle \right|=\frac{1}{N}\left|\sum_{n=1}^{N}e^{i\Delta \phi_{rel}\left(t_{n}\right)}\right|=\sqrt{{\langle \cos\Delta \phi_{rel}\left(t\right)\rangle }^{2}+{\langle \sin\Delta \phi_{rel}\left(t\right)\rangle }^{2}}$$
  where $\langle .\rangle$ denotes temporal averaging and *N* is the number of samples. A PLV value approaching 1 indicates strong phase synchronization.
  The *PLI* is defined as follows:
  (2) $$PLI=\langle \left|\mathrm{sign}(\Delta \phi_{rel}\left(t\right))\right|\rangle =\left|\frac{1}{N}\sum_{n=1}^{N}\mathrm{sign}\left(\Delta \phi_{rel}\left(t_{n}\right)\right)\right|$$
  where sign denotes signum.
  In our work, the band width is determined based on the frequency range after filtering. The *S* index quantifies synchronization between two time series [39]. Given two simultaneously recorded signals $x\left(t\right)=\left(x_{1},x_{2},\cdots ,x_{N}\right)$ and $y\left(t\right)=\left(y_{1},y_{2},\cdots ,y_{N}\right)$, delay phase-space embedding projects them into a higher-dimensional space using time-delay vectors:
  Delay phase-space vector is defined as follows:
  (3) $$\begin{array}{c} x_{n}=\left(x\left(n\right),x\left(n-\tau \right),\cdots ,x\left(n-\left(d-1\right)\tau \right)\right)\\ y_{n}=\left(y\left(n\right),y\left(n-\tau \right),\cdots ,y\left(n-\left(d-1\right)\tau \right)\right) \end{array}$$
  where *d* is the embedding dimension and τ the time delay.
  Let $r_{n,j}$ and $s_{n,j}\left(j=1,\cdots ,k\right)$ denote the time indices of the *k* nearest neighbors of $x_{n}$ and $y_{n}$. The mean Euclidean distance of $x_{n}$ to its *k* nearest neighbors is
  (4) $$R_{n}^{\left(k\right)}\left(X\right)=\frac{1}{k}\sum_{j=1}^{k}{\left|x_{n}-x_{r_{n,j}}\right|}^{2}$$
  Additionally, *Y*-conditioned mean squared Euclidean distance can be obtained:
  (5) $$R_{n}^{\left(k\right)}\left(X|Y\right)=\frac{1}{k}\sum_{j=1}^{k}{\left|x_{n}-x_{s_{n,j}}\right|}^{2}$$
  Then, the *S* index is defined as follows:
  (6) $$S^{\left(k\right)}\left(X|Y\right)=\frac{1}{N}\sum_{n=1}^{N}\frac{R_{n}^{\left(k\right)}\left(X\right)}{R_{n}^{\left(k\right)}\left(X|Y\right)}$$
  An *S* index close to 1 indicates strong synchronization between *x* and *y*.
- Effective Brain Connectivity
  To assess directional influence between signals, Granger Causality (GC) determines whether past values of signal *x* improve prediction of signal *y*, indicating that *x* influences *y* [40,41]. Partial Directed Coherence (PDC) extends this by evaluating such influence across frequency bands [42].
  A Multivariate Autoregressive (MAR) model with *M* channels and order *p* is defined as follows:
  (7) $$\left(\begin{array}{c} x_{1}\left(k\right)\\ \vdots \\ x_{M}\left(k\right) \end{array}\right)=\sum_{r=1}^{p}A_{r}\left(\begin{array}{c} x_{1}\left(k-r\right)\\ \vdots \\ x_{M}\left(k-r\right) \end{array}\right)+\left(\begin{array}{c} \varepsilon_{1}\left(k\right)\\ \vdots \\ \varepsilon_{M}\left(k\right) \end{array}\right)$$
  where $A_{1},A_{2},\cdots ,A_{p}$ are M∗M coefficient matrix, and $\varepsilon_{i}\left(k\right)$ denotes residuals. $x_{i}\left(k\right)$ represents the vector of EEG signals for channel *i* at time *k*. In the MAR model, the value of each time series is expressible as a function of its own previous values and past values of all other time series involved.
  To compute GC, let M=1 and M=2 denote models using only $x_{a}$ and both $x_{a}$ and $x_{b}$, respectively. The GC from $x_{b}$ to $x_{a}$ is defined as follows:
  (8) $$GC_{X_{b}\to X_{a}}=\ln\frac{Var(\varepsilon_{a,M=1})}{Var(\varepsilon_{a,M=2}+\varepsilon_{b,M=2})}$$
  By applying the Fourier transform to the MAR coefficients, the frequency-domain representation is obtained:
  (9) $$A\left(f\right)=\sum_{r=1}^{p}A\left(r\right)e^{-i2\pi fr}$$
  The transfer function matrix H(f) is then defined as follows:
  (10) $$H\left(f\right)={\bar{A}}^{-1}\left(f\right)={\left[I-A\left(f\right)\right]}^{-1}$$
  Let $\bar{A}\left(f\right)=\left[{\bar{a}}_{1}\left(f\right){\bar{a}}_{2}\left(f\right)\cdots {\bar{a}}_{M}\left(f\right)\right]$, with ${\bar{a}}_{ij}\left(f\right)$ being the i,j-th element of $\bar{A}\left(f\right)$, ${\bar{a}}_{i}\left(f\right)$ being the *i*-th column of the matrix $\bar{A}\left(f\right)$. The PDC from channel *j* to channel *i* is given by the following:
  (11) $$PDC\left(f\right)=\pi_{ij}\left(f\right)=\frac{{\bar{a}}_{ij}\left(f\right)}{\sqrt{{\bar{a}}_{j}^{H}\left(f\right)\;{\bar{a}}_{j}\left(f\right)}}$$
  For each sample, PDC matrices were computed across multiple frequencies and averaged to form the final representation.
- Network Properties
  We extracted five connectivity indicators—PLV, PLI, GC, PDC, and *S* index—across three frequency bands (theta, alpha and beta), yielding 15 weighted connectivity matrices per trial (5 indicators × 3 bands). To derive global network features, each weighted matrix was thresholded at 130 linearly spaced values between 0 and 1. For each threshold, values above the threshold were binarized to form adjacency matrices, from which four graph-theoretic metrics were computed: global efficiency, local efficiency, clustering coefficient, and node degree. These metrics were aggregated (summed and averaged) across thresholds, resulting in 10 selected features per matrix, and a total of 150 global features (3 bands × 5 indicators × 10 features) for the dataset with 21 EEG channels. To capture local connectivity patterns and address potential feature sparsity, we also extracted the upper triangular elements (excluding the diagonal) from each original 21 × 21 weighted connectivity matrix, yielding 21 values per matrix and 315 local features in total (15 matrices × 21 values).
  The same processing pipeline was applied to Dataset 2 containing 32 EEG channels, ensuring consistency across datasets. Due to the increased number of nodes, the dimensionality of derived features increased accordingly. For each thresholded binary matrix, 35 graph-theoretic features were selected (expanding the metric set beyond the initial four), resulting in 525 global features (3 bands × 5 indicators × 35 features). Similarly, extracting the upper triangular values from each 32 × 32 weighted matrix yielded 496 values per matrix, leading to 7440 local connectivity features (15 matrices × 496 values).

#### 3.3.2. STFT

In this study, the time–frequency representation of EEG signals was obtained using the short-time Fourier transform (STFT), following the methodology described in previous literature [43,44]. Raw EEG signals from each channel were segmented and transformed using the Blackman window.

The STFT of a signal x(t) is defined as follows:

(12) $$X_{STFT}\left(t,\omega \right)=\sum_{m=-\infty }^{\infty }x\left(m\right)w\left(m-t\right)e^{-j\omega m}$$

where w(m−t) denotes the window function applied at time *t*. The resulting spectrogram is given by the following:

(13) $$S\left(t,\omega \right)=|X_{STFT}{\left(t,\omega \right)|}^{2}$$

We computed STFT for EEG each channel using a segment length ΔT=5 s. The Blackman window function with length M=128 was applied, defined as follows:

(14) $$\begin{array}{c} w\left(n\right)=0.42-0.5\cos\left(\frac{2\pi n}{M-1}\right)+0.08\cos\left(\frac{4\pi n}{M-1}\right),\\ 0\leq n\leq M-1 \end{array}$$

STFT spectrograms S(t,Ω) were computed on a per-second basis and averaged over non-overlapping 5 s windows to construct a single sample. To ensure consistent temporal sampling, adjacent samples were extracted at 1 s intervals. Following the approach of Acı et al. [20], the averaged spectrograms were converted to decibel (dB) scale to enhance discriminative power. For Dataset 1, which contains recordings from 7 EEG channels, each spectrogram comprised 80 frequency bins, yielding a feature vector of 7×80=560 dimensions per sample. To ensure methodological consistency, the same STFT-based feature extraction strategy was applied to Dataset 2, which includes a denser array of 32 EEG channels. Accordingly, the dimensionality of each sample increased to 32×80=2560 features, reflecting the higher spatial resolution of the EEG recordings in Dataset 2.

#### 3.3.3. Statistical Feature Construction

To comprehensively characterize the EEG signals, we employed a unified hybrid feature construction strategy that integrates spatial, spectral, temporal, and statistical information, and applied it consistently to both Dataset1 and Dataset2. The initial feature set comprised three components: brain connectivity matrices derived from five functional connectivity metrics (PLV, PLI, GC, PDC, and *S* index) across three frequency bands and ten thresholds; STFT-based spectral features extracted per channel over 80 frequency bins; and first-order difference features computed by subtracting adjacent frequency components within each channel to encode local spectral dynamics.

For Dataset 1, which includes seven EEG channels, this resulted in 560 STFT spectral features and 559 difference features per sample. These features, along with the vectorized connectivity matrices, were organized into 15 groups: one group for the connectivity features, seven for the raw STFT features (one per channel), and seven for the corresponding difference features. From each group, we computed ten statistical descriptors—including lower and upper quartiles, trimmed mean (excluding the lowest and highest 10%), minimum, maximum, standard deviation, mean square, skewness, kurtosis, and sum of square roots, yielding a total of 150 statistical features. Additionally, we extracted a single temporal dynamic feature based on the number of zero-crossings in the STFT difference signals. Together with 150 connectivity descriptors (from five metrics, three bands, and ten thresholds) and 315 raw upper-triangular connectivity matrix values (21 values from each of 15 thresholded matrices), the final consolidated feature vector for Dataset1 comprised 1735 dimensions.

The same processing pipeline was applied to Dataset 2, which contains 32 EEG channels. The increase in spatial resolution led to a substantial rise in feature dimensionality. Specifically, the number of STFT spectral features expanded to 2560 (32 × 80), and the difference features increased to 2559 (32 × 79). These features were grouped into 65 categories: 1 for the vectorized brain connectivity matrices, 32 for the channel-wise STFT features, and another 32 for their corresponding difference features. Ten statistical descriptors were again computed per group, resulting in 650 statistical features, and 1 temporal dynamic feature was derived from the difference signals as before. Given the expanded connectivity matrices (32 channels yielding 496 upper-triangular elements per matrix), the number of raw connectivity values reached 7440 across 15 thresholded matrices. In addition, the number of graph-based connectivity features grew to 525, reflecting the larger node set. Altogether, the final feature vector for Dataset 2 comprised 13,735 dimensions, following the same structural composition as Dataset1 but scaled according to the higher channel count.

### 3.4. Feature Selection

**Figure 3:**
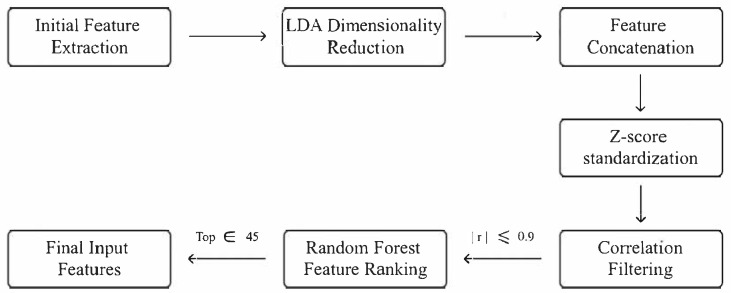
Feature selection pipeline.

To reduce redundancy and enhance the discriminative power of features, we employed a structured feature selection pipeline (illustrated in Figure 3) applied consistently across both datasets. For the Dataset 1, the original features were organized into 17 subsets, including raw connectivity matrices across three frequency bands, STFT features from seven EEG channels, and their respective difference features. Each subset was independently projected to a 2D latent space using LDA, preserving class-separability while maintaining local structure. This step yielded a 34-dimensional intermediate representation (17 subsets × 2 dimensions). The LDA-reduced features were then concatenated with 150 statistical and global graph features, excluding those derived from PLI due to low inter-class variance. The resulting 305-dimensional combined feature set was standardized via z-score normalization (zero mean, unit variance, computed from training data). To further reduce redundancy, we calculated pairwise Pearson correlation coefficients and removed features with absolute correlation above 0.9, reducing the dimensionality to fewer than 270. Subsequently, a random forest-based feature ranking (100 estimators, $max_{f}eatures{=}^{\prime }sqrt^{\prime },random_{s}tate=34$) was applied, and the top 45 most informative features were selected as input for classification. Importantly, all steps were performed exclusively on training data to prevent information leakage and ensure fair model evaluation.

The same processing strategy was applied to the Dataset 2, with adaptations reflecting its increased spatial resolution. Specifically, the original features were organized into 67 subsets, comprising raw connectivity matrices (three bands), STFT features from 32 channels, and corresponding difference features. Each subset underwent independent LDA-based dimensionality reduction to 2D, yielding a 134-dimensional intermediate representation (67 × 2). These were concatenated with 650 statistical and global graph features (excluding PLI-based features), along with 1 temporal dynamic feature, forming a combined 1205-dimensional feature vector. Following the same protocol, z-score normalization was applied, and highly correlated features ($\left|r\right|>0.9$) were discarded, resulting in fewer than 1000 features. Finally, random forest ranking with identical parameters was used to select the top 45 features for classification. All steps were again restricted to training data, ensuring robustness, consistency, and reproducibility across datasets.

### 3.5. Classifier

To comprehensively evaluate the classification performance, a SVM with a radial basis function (RBF) kernel was employed under under both intra-subject and inter-subject settings. In the intra-subject scenario, data from each participant were randomly divided into training (80%) and testing (20%) sets without overlap. The SVM was trained on the subject-specific training data, with hyperparameters *C* and gamma optimized using five-fold cross-validation, enabling the model to learn individualized decision boundaries. For inter-subject classification, two strategies were adopted. The first was a pooled training approach, where data from all subjects were aggregated and randomly split into training (80%) and testing (20%) sets, allowing the model to learn shared representations across individuals. The second was a leave-one-subject-out (LOSO) strategy, in which data from N−1 subjects were used for training and the held-out subject was used for testing, iterating until each subject had served as the test subject once. In both cross-subject settings, hyperparameter tuning was performed using five-fold cross-validation on the training set to ensure generalizable performance.

## 4. Results

### 4.1. Comparison with State-of-the-Art Methods

As summarized in Table 1, prior studies employ a variety of feature extraction and classification techniques but often suffer from inconsistent evaluation protocols and limited generalization. For example, the STFT+SVM method yields 76.45% accuracy under intra-subject validation on Dataset 1 [20], while RDWT combined with ensemble machine learning achieves 85.64% in inter-subject evaluation [45]. However, performance drops markedly under more stringent conditions: STFT+CNN reaches only 53.22% accuracy in inter-subject leave-one-trial-out validation [46], and STFT+DNN achieves just 71.10% under leave-one-subject-out evaluation [47]. On Dataset 2, conventional classifiers such as SVM, RF, EEGNet, and DGCNN exhibit significant performance variability across protocols. For instance, EEGNet drops from 85.12% to 64.84% in accuracy between intra- and inter-subject settings, and its F1 score changes from 59.64 to 64.84. Similarly, RF shows a sharp decline in F1 score from 59.56 to 45.21, reflecting the models’ sensitivity to subject variability and protocol design.

In contrast, the proposed method consistently outperforms existing approaches across both datasets and various evaluation schemes. On Dataset 1, it achieves 86.27% accuracy under standard inter-subject evaluation, surpassing the previous best of 85.64% by RDWT+Ensemble ML, along with an F1 score of 86.22, recall of 86.27, and precision of 86.40. Under the challenging leave-one-trial-out setting, the proposed method attains 67.99% accuracy and 65.21 F1 score—an absolute improvement of nearly 27.8% over STFT+CNN. Performance remains stable across different paradigms: 86.27% (inter-subject), 67.99% (leave-one-trial-out), and 72.27% (leave-one-subject-out), yielding a fluctuation of just 18.28%, significantly lower than the 32.42% variation seen in baseline methods. Even under intra-subject evaluation without data shuffling, the method achieves strong generalization with 77.61% accuracy, F1 score of 77.22, recall of 77.29, and precision of 77.22.

On Dataset 2, the superiority of our method is even more pronounced. It achieves 96.61% accuracy under intra-subject evaluation, with an F1 score of 96.56, recall of 96.60, and precision of 96.60. In inter-subject testing, it attains 94.01% accuracy, outperforming EEGNet (64.84%) and achieving an F1 score of 90.74, recall of 88.51, and precision of 93.74. Under the stringent leave-one-trial-out condition, it reaches 83.27% accuracy and 65.41 F1 score, far exceeding DGCNN’s 63.91%. Even in the leave-one-subject-out setting, where performance naturally declines due to higher variability, the method retains competitive results with 48.00% accuracy, 41.99 F1 score, 42.48 recall, and 49.74 precision. Additionally, in the non-shuffled intra-subject evaluation, it achieves 92.14% accuracy with an F1 score of 92.14, recall of 92.14, and precision of 92.14, indicating strong resistance to overfitting and enhanced temporal generalization.

We evaluated the impact of sample overlap on classification performance using our proposed method. As shown in Table 2, introducing overlap between adjacent samples led to notable accuracy improvements across all subjects.

The results demonstrate a clear performance gain with increased overlap duration across both datasets. For Dataset 1, applying a 3 s overlap (approximately 60% overlap rate) yields the highest average accuracy (87.09%), outperforming the 1 s (82.48%) and non-overlapping (80.30%) configurations. Notably, the F1 score, recall, and precision also improve substantially, increasing from 80.33%, 80.34%, and 80.53%, respectively, without overlap, to 87.72%, 87.71%, and 87.76% with a 3 s overlap. These results suggest that overlapping segmentation enhances temporal continuity and increases sample diversity, thereby boosting classification robustness. A similar trend is observed in Dataset 2, where the average accuracy for Subjects 1–10 increases from 96.61% in the non-overlapping scenario to 99.07% with a 1 s overlap and reaches 99.61% with a 3 s overlap. The recall improves accordingly, rising from 93.37% to 99.31%. For Subjects 11–20, the F1 score improves from 94.77% without overlap to 99.21% with a 3 s overlap, while precision increases from 97.35% to 99.26%. It is noteworthy that under the 3 s overlap setting, 15 out of 20 subjects achieved 100% classification accuracy, highlighting the method’s high consistency and reliability across individuals.

### 4.2. Effect of Window Size on Performance

**Figure 4:**
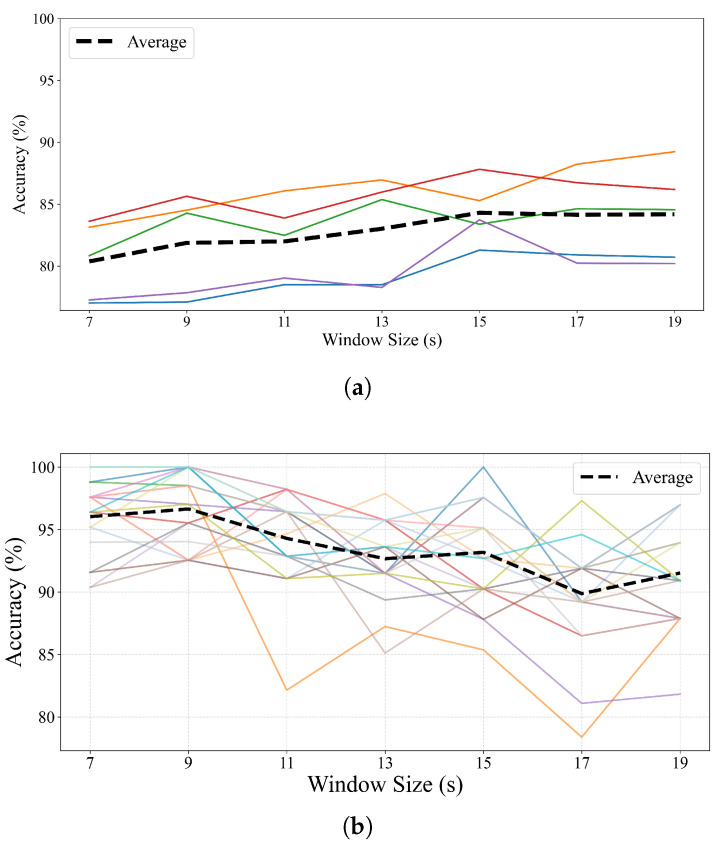
Classification accuracy across different temporal window sizes for each subject: (**a**) Dataset1; (**b**) Dataset2. The dashed black line indicates the average accuracy across all subjects at each window size. Colored lines represent individual subjects’ accuracy trends.

Figure 4 illustrates the classification performance across different temporal window sizes for both datasets. For Dataset 1 (Figure 4a), the overall trend shows a gradual increase in accuracy as the window size extends from 7 to 19 s. The average accuracy improves from approximately 80.1% at 7 s to 85.6% at 19 s, suggesting that longer windows capture more stable EEG dynamics, which enhances feature reliability. However, subject-specific responses vary, with some reaching performance saturation or slight declines beyond 15 s, likely due to diminishing returns from fewer available segments. Statistical analysis using one-way ANOVA confirms that the differences in classification performance across window sizes are significant for Dataset 1, with a *p*-value of $7.19\times 10^{-5}$, indicating that window size has a statistically meaningful effect on classification accuracy.

In contrast, Dataset 2 (Figure 4b) exhibits a different trend: the highest average accuracy occurs at 9 s, after which performance slightly declines with longer windows. Although individual subjects vary considerably, many follow a similar pattern, peaking early (7–11 s) and then showing performance degradation beyond 13 s. This drop may be attributed to the reduced number of training instances when longer windows are used, particularly in a high-dimensional setting with 32-channel data, where overfitting becomes more likely under sparse training conditions. This trend is also statistically significant, as evidenced by a *p*-value of $2.71\times 10^{-7}$ from the one-way ANOVA, reinforcing the impact of window size on model performance.

These findings suggest that while longer windows may improve temporal feature stability, the optimal window size depends on dataset characteristics such as channel count, trial duration, and inter-subject variability. In our case, 15 s yields a balanced trade-off between accuracy and sample count for Dataset 1, whereas shorter windows (7–9 s) are more effective for Dataset 2.

### 4.3. Method Performance Without Data Shuffling

Recent studies have emphasized that conventional EEG classification protocols, which involve random shuffling of samples during the train–test split, may lead to overly optimistic results due to temporal autocorrelation between adjacent samples [48]. In particular, when training and testing samples are drawn from closely recorded time windows, models may inadvertently exploit low-level temporal dependencies rather than learning meaningful neurophysiological patterns. To address this issue, we conducted a more rigorous evaluation by strictly preserving the temporal order of the data when splitting the training and testing sets. To avoid data leakage, we ensured that there was no overlap between the final training sample and the first test sample. Specifically, we assigned the first 85% of samples (based on temporal order) to the training set, and the remaining 15% to the test set. To further reinforce temporal separation, we discarded the first 17 samples of the test segment, introducing a time buffer between the two sets. This evaluation protocol serves as a compromise between leave-one-out and block-wise cross-validation, striking a balance between temporal independence and data availability.

The classification results under this strict non-shuffled setting are summarized in Table 3. For Dataset 1, the average accuracy across all five subjects reached 77.61%, with subject-level averages ranging from 72.99% to 85.68%. Notably, Subject 2 and Subject 4 exceeded 80% average accuracy. Among the 23 repeated runs per subject, 10 achieved accuracy above 80%, while only 4 fell below 70%. These results confirm that even without temporal overlap during train–test splitting, the proposed framework retains strong discriminative power and temporal generalizability. For Dataset 2, the performance was consistently high across all 20 subjects. The average classification accuracy ranged from 90.96% to 99.23%, with most subjects achieving accuracies well above 95%. In particular, 10 subjects reached a maximum accuracy of 100.00%, and several maintained minimum accuracies above 90%, even across multiple non-shuffled runs. Such robustness under strict evaluation indicates that our method not only scales effectively to higher-density EEG setups but also maintains temporal stability and generalization across trials without requiring artificial shuffling.

Overall, these results highlight the reliability and flexibility of our method under realistic deployment conditions, where temporal continuity must be preserved to ensure causal and practical application.

## 5. Discussion

In this section, we analyze the discriminative characteristics of brain connectivity under different mental states, summarize the most informative features selected across subjects, and critically evaluate the potential causes of "excellent" performance reported in prior work.

### 5.1. Discriminative Patterns of Granger Causality

**Figure 5:**
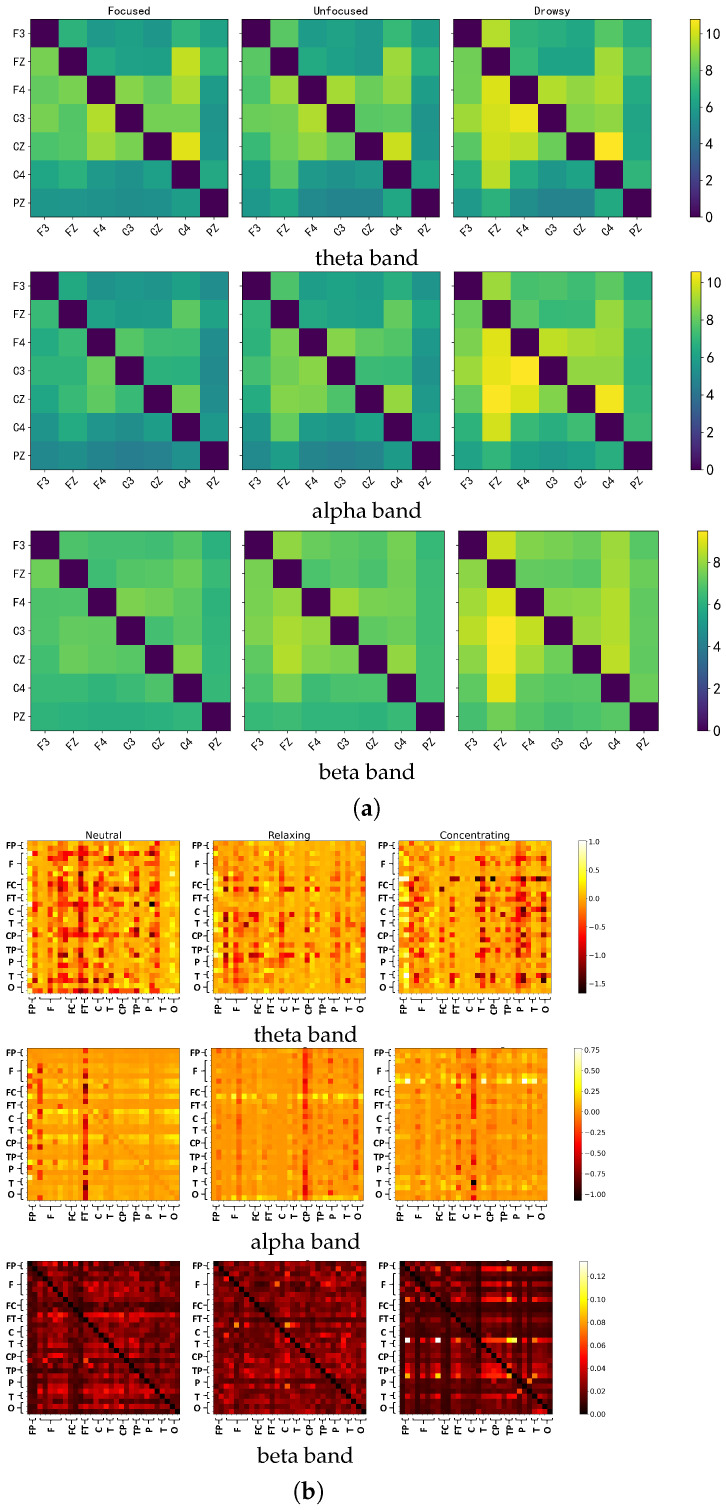
Granger Causality (GC) connectivity matrices across three frequency bands for the two datasets. (**a**) Dataset 1: averaged GC matrices (7 channels) under focused, unfocused, and drowsy states in the theta, alpha, and beta bands. (**b**) Dataset 2: corresponding GC matrices (32 channels) under neutral, relaxing, and concentrating states across the same frequency bands. Each matrix represents the average causal influence across all subjects under the given condition and frequency.

To explore the discriminative power of brain connectivity features, we focused on GC, which outperformed other connectivity indicators in preliminary evaluations. For both Dataset 1 and Dataset 2, we computed the average GC matrices across all subjects, three mental states (focused/neutral, unfocused/relaxing, and drowsy/concentrating), and three frequency bands (theta, alpha, beta), resulting in 45 matrices per dataset. The results are visualized in Figure 5a,b, with values scaled for visualization.

Across both datasets, GC patterns exhibit distinct modulation with cognitive states, particularly in the theta and alpha bands. In Dataset 1 (Figure 5a), drowsy states elicit stronger and more widespread causal interactions, especially across centro-parietal regions (e.g., C4–PZ), while focused states demonstrate more localized, sparser connectivity. Dataset 2 (Figure 5b) extends these findings to a finer spatial scale. For instance, in the theta band, relaxing and neutral conditions show dense GC activity across fronto-parietal and occipital sites, while the concentrating state suppresses many long-range interactions. This contrast is especially evident in the beta band, where focused attention consistently yields sparser connectivity in both datasets, suggesting that attentional engagement is accompanied by more selective, top-down modulation of predictive brain dynamics.

**Figure 6:**
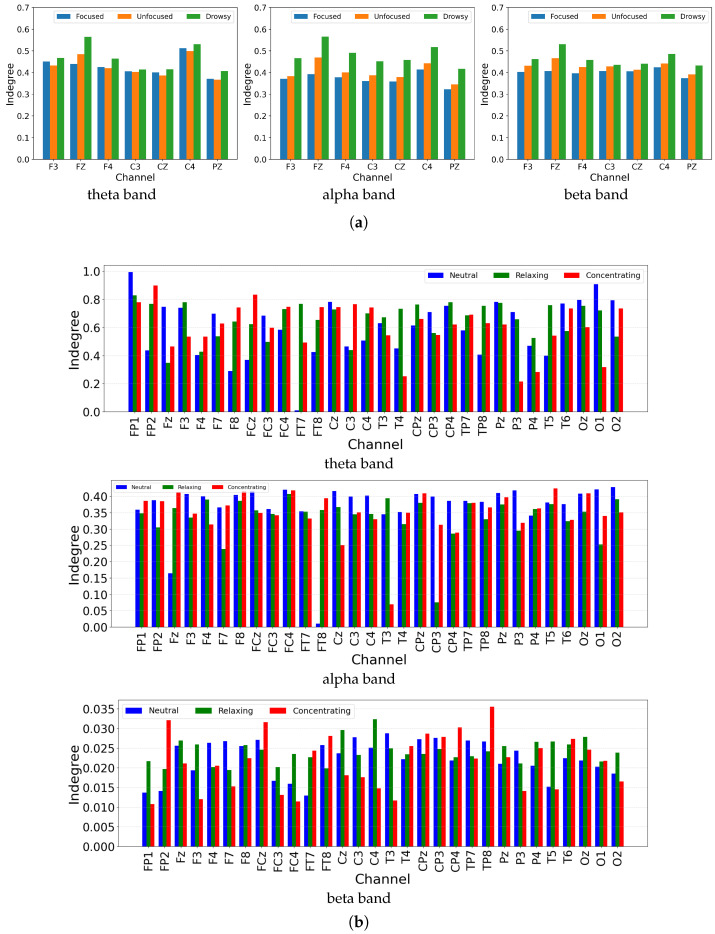
Average indegree of Granger Causality matrices under different mental states and frequency bands for two datasets. (**a**) Dataset 1; (**b**) Dataset 2.

To further quantify the effect of mental attention states on causal brain dynamics, we computed the average GC indegree (i.e., total incoming information flow) for each channel across different mental conditions and frequency bands. The results are shown in Figure 6. In Dataset 1, the drowsy state consistently exhibits higher indegree values across most channels in the theta, alpha, and beta bands, followed by the unfocused and focused states. This suggests that decreased attentional engagement is associated with more globally distributed information flow, potentially reflecting less top-down modulation and increased bottom-up activity. The focused state, by contrast, shows reduced indegree across the board, implying a more selective and efficient information processing regime. One-way ANOVA confirmed that these state-related differences were highly significant across channels in all three bands. For example, in the theta band, we observed F3 ($p=2.85\times 10^{-37}$), FZ ($p=3.12\times 10^{-196}$) and PZ ($p=3.53\times 10^{-45}$). In the alpha band, F3 ($p=1.32\times 10^{-274}$), FZ($p<10^{-300}$) and PZ ($p=2.80\times 10^{-269}$) all reached significance. The beta band similarly showed F3 ($p=2.79\times 10^{-100}$), FZ ($p=2.09\times 10^{-301}$) and C3 ($p=2.25\times 10^{-48}$). In Dataset 2, which includes 32 EEG channels, a similar trend is observed with greater spatial resolution. The neutral and relaxing states generally present higher indegree values than the concentrating state, particularly in the theta and alpha bands. This pattern is most evident in frontal (e.g., FP1, F3) and parietal (e.g., P3, P4) regions, highlighting a broader and more integrative cortical network under relaxed or resting conditions. In contrast, the concentrating state demonstrates selective suppression of incoming information, likely supporting goal-directed cognitive control. In the beta band, although overall GC strength is lower, the concentrating condition still shows relatively reduced indegree compared to the other states, supporting the consistency of this attentional modulation across frequency scales. One-way ANOVA again verified significant state effects: in the theta band, electrodes such as FP2 ($p=2.71\times 10^{-2}$), FC3 ($p=7.55\times 10^{-8}$), and FT7 ($p=7.44\times 10^{-3}$) differed across conditions; in the alpha band, FP1 ($p=1.86\times 10^{-4}$), F4 ($p=2.41\times 10^{-12}$), and FCz ($p=2.75\times 10^{-5}$) were significant; and in the beta band, FP1 ($p=3.76\times 10^{-36}$), F3 ($p=3.12\times 10^{-14}$), and CP3 ($p=1.99\times 10^{-17}$) all showed robust differences. These statistical results reinforce the modulatory role of attention on causal information flow across both datasets, frequency bands, and cortical regions.

These observations confirm that Granger Causality not only captures interpretable patterns of cognitive modulation but also scales effectively across datasets, making it a strong candidate for cross-condition and cross-subject brain state decoding.

### 5.2. Re-Evaluating “Excellent” Performance in Previous Work

In prior studies, notably Çiğdem İnan Acı et al., exceptionally high classification accuracy was reported. However, their method involved a 15 s moving average of the spectrogram, leading to a 14 s overlap between adjacent samples. Although they reported using 6000 training and 1500 testing samples per subject, such sample sizes are theoretically unattainable without extensive overlap or synthetic resampling, especially given a total experiment duration of only 65 min.

**Figure 7:**
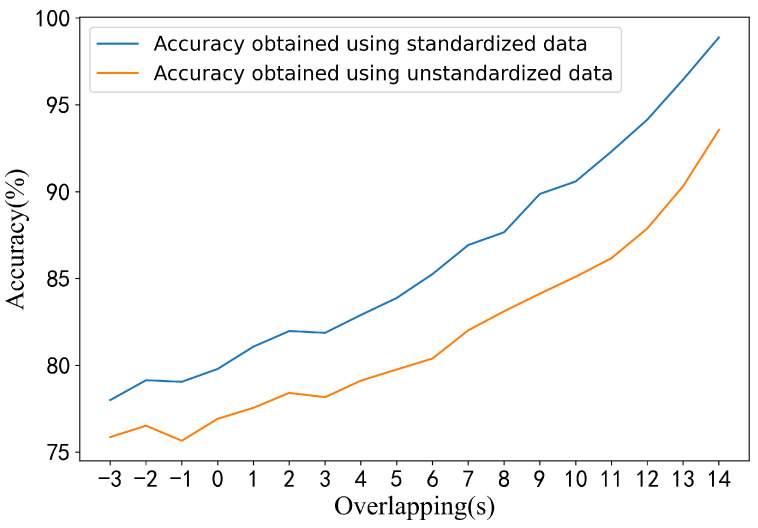
Accuracy comparisons under different overlapping when replicating method proposed by Çiğdem İnan Acı et al.

To evaluate the effect of such overlapping and data partitioning practices, we approximately replicated their setup under different overlap settings. When samples were assigned to training and test sets randomly (i.e., with potential temporal leakage), performance increased with overlap and matched the original study’s results under a 14 s overlap. However, when we applied a temporally ordered, non-shuffled split and standardized the data, performance significantly declined (shown in Figure 7). Specifically, average accuracy dropped to 53.65%, compared to the 77.61% achieved using our proposed method under a strict no-shuffling protocol (Section 4.3).

These findings suggest that the high performance reported in previous work may have resulted from data leakage due to excessive overlapping and random partitioning. Our results underscore the importance of using temporally disjoint training and testing sets for rigorous EEG classification benchmarking.

## Figures and Tables

**Table 1:** Comparative analysis of classification performance across different experimental paradigms. Values represent mean accuracy (%), F1 score, recall, and precision.

Reference	Experimental Condition	Dataset	Accuracy (%)	F1 Score	Recall	Precision
STFT+SVM [20]	Intra-subject	Dataset 1	76.45	/	/	/
RDWT+Ensemble ML [45]	Inter-subject	Dataset 1	85.64	/	/	/
STFT+CNN [46]	Inter-subject, leave-one-trial-out	Dataset 1	53.22	/	/	/
STFT+DNN [47]	Leave-one-subject-out	Dataset 1	71.10	/	/	/
SVM [23]	Intra-subject/Inter-subject	Dataset 2	78.71/62.28	54.82/43.35	/	/
RF [23]	Intra-subject/Inter-subject	Dataset 2	80.55/56.03	59.56/45.21	/	/
EEGNet [23]	Intra-subject/Inter-subject	Dataset 2	85.12/64.84	59.64/64.84	/	/
DGCNN [23]	Intra-subject/Inter-subject	Dataset 2	82.14/63.91	47.59/43.65	/	/
Proposed Method	Intra-subject	Dataset 1	84.30	83.90	84.00	84.30
		Dataset 2	96.61	94.77	93.34	97.35
	Inter-subject	Dataset 1	86.27	86.22	86.26	86.40
		Dataset 2	94.01	90.74	88.51	93.74
	Inter-subject, leave-one-trial-out	Dataset 1	67.99	65.31	68.05	72.44
		Dataset 2	83.27	62.14	65.32	73.21
	Intra-subject, no shuffling	Dataset 1	77.61	77.22	77.92	81.37
		Dataset 2	92.14	82.15	81.39	88.33
	Leave-one-subject-out	Dataset 1	72.27	70.75	72.37	71.03
		Dataset 2	48.00	41.99	42.48	49.74

**Table 2:** Classification accuracies (%) under different overlap durations for Dataset 1 and Dataset 2.

Dataset	Subject	−1 s	1 s	3 s	Subject	−1 s	1 s	3 s
Dataset 1	1	79.41	80.40	84.61	2	83.18	85.31	88.72
	3	80.33	80.19	85.80	4	82.88	87.21	92.01
	5	75.68	79.29	84.31	–	–	–	–
	**Average**	80.30	82.48	87.09	**F1 score**	80.33	82.39	87.72
	**Recall**	80.34	82.36	87.71	**Precision**	80.53	82.55	87.76
Dataset 2	1	99.11	100.00	100.00	2	96.43	98.80	100.00
	3	93.75	98.19	99.09	4	98.21	100.00	99.09
	5	99.11	98.80	100.00	6	95.54	97.59	100.00
	7	90.18	99.40	96.37	8	97.32	100.00	100.00
	9	99.11	100.00	100.00	10	92.86	96.99	100.00
	11	93.75	98.80	99.40	12	91.07	97.59	99.40
	13	100.00	100.00	99.70	14	98.21	100.00	100.00
	15	99.11	99.40	100.00	16	98.21	98.80	99.70
	17	96.43	96.99	99.40	18	98.21	100.00	100.00
	19	99.11	100.00	100.00	20	96.43	100.00	100.00
	**Average**	96.61	99.07	99.61	**F1 score**	94.77	98.12	99.21
	**Recall**	93.37	97.69	99.31	**Precision**	97.35	98.87	99.26

**Table 3:** Classification accuracies (%) without shuffling during train–test split, by dataset and subject.

Dataset	Subject	Avg	Max	Min	Subject	Avg	Max	Min
Dataset 1	1	72.99	87.68	54.16	2	80.79	98.66	57.21
	3	73.90	91.66	50.72	4	85.68	90.57	81.59
	5	75.97	84.78	66.66	–	–	–	–
Dataset 2	1	96.22	98.59	90.77	2	98.92	100.00	97.18
	3	94.84	100.00	89.55	4	96.27	98.51	94.03
	5	98.15	100.00	95.38	6	98.57	100.00	95.77
	7	95.75	100.00	84.51	8	93.07	98.46	88.73
	9	96.69	98.51	94.03	10	93.25	98.59	89.55
	11	91.13	96.92	86.57	12	96.02	98.51	90.14
	13	95.89	98.51	93.85	14	95.10	98.59	90.77
	15	99.23	100.00	96.92	16	96.18	100.00	87.69
	17	93.68	97.01	89.23	18	95.56	100.00	93.85
	19	90.96	97.18	83.08	20	98.20	100.00	95.77

## Data Availability

The two datasets used in our study are publicly available and can be downloaded from https://www.kaggle.com/datasets/inancigdem/eeg-data-for-mental-attention-state-detection (accessed on 12 October 2023) (Dataset 1) and https://github.com/XJTU-EEG/MEMA (accessed on 21 May 2025) (Dataset 2), subject to their respective usage terms and licensing agreements.

## Abbreviations

The following abbreviations are used in this manuscript:

EEG	Electroencephalography
ICA	Independent Component Analysis
STFT	Short-Time Fourier Transform
PLV	Phase Locking Value
PLI	Phase Lag Index
GC	Granger Causality
PDC	Partial Directed Coherence
SVM	Support Vector Machine
